# Trajectories of pain predict disabilities affecting daily living in arthritis

**DOI:** 10.1111/bjhp.12364

**Published:** 2019-04-07

**Authors:** Richard J. E. James, David A. Walsh, Eamonn Ferguson

**Affiliations:** ^1^ School of Psychology University of Nottingham UK; ^2^ Arthritis UK Pain Centre School of Medicine University of Nottingham UK; ^3^ NIHR Nottingham Biomedical Research Centre University of Nottingham UK; ^4^ Academic Rheumatology School of Medicine University of Nottingham UK

**Keywords:** activities of daily living, arthritis, disability, pain, personalized medicine

## Abstract

**Purpose:**

To examine the interplay between pain and disability in arthritis when adjusting for patient heterogeneity in pain progression. There is consistent evidence to suggest that people experience osteoarthritis *heterogeneously*, with subgroups of people having different trajectories of pain. However, at present it is unclear how these pain trajectories are related to functional disability. We ask the question: Do levels of disability track changes in pain across different pain trajectories?

**Methods:**

Secondary analysis of a subset (*n* = 889) from a cohort of older English adults, representative of the general population (the English Longitudinal Study of Ageing). The relationship between pain and functional disability was compared in three domains of disability: mobility, activities of daily living (ADL) and instrumental ADL. These represent increasingly complex forms of self‐care required for independent living. Data analysis compared the heterogeneous analysis of pain (different trajectories) and disability compared to treating pain as a simpler homogenous construct.

**Results:**

On a population level, pain was significantly positively correlated with increased disability in all three domains, and the relationship remained stable over time. However, when heterogeneity was examined respondents whose pain improved did not show a corresponding improvement in disability in two domains (ADL and mobility).

**Conclusions:**

These findings highlight how, for some people, alleviating pain, the main symptom of arthritis, might not prevent the persistence or progression of disability. Even when pain improves, further interventions that improve disability are likely to be required.

**Statement of contribution**

***What is already known on this subject?***

- Pain and functional limitation in daily living are common symptoms of arthritis.
- Arthritis pain is heterogeneous – there are trajectories of people whose pain gets better or worse.
- However, to date no study has looked at the relationship between trajectories of arthritis pain and functional disability outside of the minority of people with rheumatoid arthritis.

***What does this study add?***

- Treating pain as heterogeneous explained disability better than treating pain as a single entity.
- Respondents in a trajectory of worsening pain reported functional disability in two domains (mobility and activities of daily living) also got worse over time.
- People in a trajectory of decreasing pain over time did not experience a reduction in disability, despite pain being the most common reason for why people limit their daily functioning.
- This suggests further intervention is required for people with arthritis, even when the most visible symptoms have been alleviated.

## Background

Osteoarthritis (OA) is the commonest cause of arthritis‐related pain and disability, particularly affecting older adults, with growing evidence that there are reliable subgroups of people with arthritis who have very different experiences of their pain over time (James, Walsh, & Ferguson, 2018). Thus, the experience of pain is *heterogeneous* and not accounting for this heterogeneity may result in missing clinically relevant associations for some subgroups. This speaks to a personalized medicine agenda where subgroups are stratified based on their psychological characteristics as much as genetic stratification (Ferguson, 2013). There have been a number of studies looking at trajectories of disability, including some simultaneously modelling pain. Most of these have been studied in the context of interventions, such as physiotherapy and total knee replacement (Dowsey, Smith, & Choong, 2015; Lee *et al*., 2018; Lenguerrand *et al*., 2016) and so look at the improvement in disability in response to some form of treatment. However, we know little about the changing association between arthritic pain and disability in the absence of a surgical or physiotherapy intervention, which are often only instigated when the disease state is advanced. Therefore, in this paper we explore the natural history of the association between pain and disability, with disability measured using the activities of daily living (ADL), using a multi‐wave panel data set.

A previous study looked the relationship between trajectories of disability and their association with pain in early rheumatoid arthritis (Norton *et al*., 2013). To date, however, the relationship between disability and the heterogeneity of pain progression has not been examined, and not among the older population with arthritis more generally. Rheumatoid arthritis comprises a small minority of arthritis cases (Humphreys *et al*., 2013; Litwic, Edwards, Dennison, & Cooper, 2013), for whom specific, disease‐modifying treatments are available. For most people with arthritis, including those with OA, disease modification is not possible, and treatment is primarily symptomatic. Arthritic pain progression is heterogeneous (James *et al*., 2018), and there is evidence of a subgroup reporting significant arthritis‐related pain that improves. We therefore should see an improvement in disability, if pain and disability track each other in a simple linear fashion. We therefore here explore for the first time if the variation observed in arthritic pain progress maps onto disability.

Studies examining the number of pain trajectories for arthritis pain commonly report 3–5 distinct trajectories, which differ in terms of initial levels of pain and the subsequent speed and direction of change in pain over time (see James *et al*. (2018) for a review). To date, there are no data on how these trajectories might relate to functional behavioural outcomes such as disability. We examine whether patients with different pain trajectories have concordant changes in their behaviour in daily life. There is increasing evidence that arthritis is not necessarily a progressive worsening condition, with pain recession as well as progression (James *et al*., 2018). It is therefore particularly important to understand whether, given many cite pain as a reason for the limitations in ADL, a reduction in pain is associated with improvements in ADL. To address this gap in the literature, the second analysis in this study examines how disability differs across four trajectories of arthritic pain progression that have previously been identified in the English Longitudinal Study of Ageing (ELSA) cohort (James *et al*., 2018). These differed in initial pain and whether they changed over time.

### Disability and ADL

This paper looks at the effects of pain trajectories on ADL. When pain is treated as a single homogenous construct, functional disability is a major consequence of arthritis, with people citing pain as a cause for limiting their ADL (Verbrugge & Juarez, 2006). However, it is not known whether the impact of pain on disability is the same for all people, especially between those who have very different experiences of pain progression over time (James *et al*., 2018). From the perspective of a personalized medicine agenda, understanding the effects of this heterogeneity is crucial (Ferguson, 2013). Therefore, this paper reports two analyses (one that treats pain progression as homogeneous and one as heterogeneous) that explore the relationship between pain and disability over seven waves of the ELSA.

The relationship between trajectories of pain and disability in ADL poses an interesting test for theoretical models commonly used in health psychology such as the Health Beliefs Model (Janz & Becker, 1984) and the Theory of Planned Behavior (Ajzen, 1991). Both models place considerable emphasis on individual perceptions of actions. These models also suggest there should be a simple linear association between pain (severity) and disability, as pain increases so should disability and as pain decreases levels of disability should improve. As pain appears to be one of the main contributing factors in limiting ADL (Verbrugge & Juarez, 2006), reducing pain ought to lead to an increase in perceived behavioural control or a reduction in perceived severity and thus lead to an increase in activity. While this may be the case when a disease is treated as homogeneous, it does not mean it will be the case when heterogeneity in terms of pain trajectories in the patient group is considered. At present, models such as the Health Beliefs Model and Theory of Planned Behavior do not account for patient heterogeneity specifically.

The ELSA cohort collects data about difficulties related to arthritis disability across three domains: mobility, ADL, and instrumental ADL (IADL), which are measured at each wave. These cover different activities that allow older adults to remain independent; mobility focuses on the performance of certain actions (e.g., climbing stairs), whereas ADL and IADL, respectively, focus on basic (e.g., bathing) and complex (e.g., managing money) activities that constitute self‐care. We first take a homogenous approach to understanding pain and examine the associations between pain and disability. This aims to establish the magnitude of the association between pain and disability, and its stability over time. The second analysis elaborates on this and explores these associations within a heterogeneous framework, by examining the temporal changes in disability between groups of patients assigned to different trajectories of pain progression, and tests whether these groups have different levels of difficulty in their daily lives. We have used a growth model framework, which assesses whether these groups have different levels of pain at baseline (adjusting for age and sex), and whether respondents in different trajectories report greater changes in their levels of disability over the seven waves of the ELSA.

## Method

### Participants

We analysed a subsample of 889 arthritic respondents from the ELSA, a representative cohort of older adults (50+) and their spouses, tracked over seven waves, covering a period from 2002 to 2015 with interviews every other year. Respondents are interviewed about their health, cognitive function, financial status, and social lives. The data are publicly available from the UK Data Archive (Marmot *et al*., 2016). The analysis was restricted to a subsample reporting a doctor's diagnosis of arthritis between 1998 and 2002, and participated in the ELSA at wave 1. Respondents were excluded if:

1. They reported an arthritis diagnosis in 2002 at wave 2 (collected 2004–2005); this was because their diagnosis was assumed to occur after their wave 1 interview.
2. They reported comorbid arthritis and cancer at wave 1. Previous analyses using these data demonstrated that arthritis and cancer display distinct pain trajectory patterns (James *et al*., 2018).

Missing data from the mobility, ADL, and IADL section of the interview were negligible (<0.1%). All respondents had demographic data, 883 completed the disability section of the ELSA interview at wave 1 (99.3%) and 397 of the 889 completed the disability items at wave 7. The sample combined respondents with OA, rheumatoid arthritis, and other forms of arthritis.

### Measurements

Respondents were shown two flashcards, one with the activities related to mobility and one with the ADL and IADL activities. Respondents were asked if, due to a health (or memory in addition for the ADL/IADL activities) problem, they had any difficulties with the activities listed on the flashcard. The questions from each domain are reported in the Table S1. Total scores were calculated for each of these disability measures by summing the items (descriptive statistics and Cronbach's α are reported in Table S2). Pain was assessed using two questions, asking first whether respondents were often troubled by pain (yes/no) and if so, was it mild, moderate or severe. These two items were combined to give a four‐point (0 = not troubled by pain, 3 = severely troubled by pain) measure of pain.

### Trajectory assignment

Patients were assigned to one of four trajectories of arthritic pain progression previously identified in the ELSA sample (James *et al*., 2018), from waves 1 to 7, with data collection covering the period 2002–2015. Trajectory assignment was done on the basis of a latent class growth analysis of pain data from all seven waves. The model had high classification accuracy (entropy = 0.85). The first group (‘Low or no chronic pain’, *n* = 381) reported minimal pain at wave 1 that did not change over time. The second group (‘increasing chronic pain’, *n* = 143) began at a similar point to the low or no chronic pain trajectory, but showed pain progression over the repeated measurements. The third group (‘decreasing chronic pain’, *n* = 147) started off in significant pain but improved over time. The final group (‘Severe fluctuating chronic pain’, *n* = 218) began in considerable pain, improving slightly over the study but getting better and worse with significant higher order growth factors.

### Statistical modelling

Growth models were used to look at the trajectories of change across the three different domains of disability, accounting for covariates and either incorporating trajectories of pain (modelling for heterogeneity) or not. Age (*z* scored) and sex (0 = male, 1 = female) were included as covariates. For models incorporating heterogeneity, the trajectories of pain were dummy coded, with the low or no chronic pain trajectory used as the reference. Missing data were accounted for using a pattern mixture model procedure (Little, 1993). This accounts for data not missing at random, in which cases are subdivided into groups based on their dropout patterns. Sensitivity analyses were conducted excluding respondents that were known to have died during the course of the ELSA study; mortality is only partially known across the cohort as the most recent mortality data were collected at wave 5. Excluding participants known to have died did not affect the model (Table S3). As a further sensitivity analysis, the pattern mixture model without trajectories was compared against a model comprising cases with complete data (Table S4). The estimates across the models were similar, but in the pattern mixture model age was associated with a higher slope and thus greater growth in disability, but was not in the *listwise* model. This is likely because older participants would be more likely to drop out over the course of the ELSA, due to infirmity and mortality. To assess the models, indices of fit transformed from the chi‐square statistic (CFI > 0.95, TLI > 0.95, SRMR < 0.05, RMSEA < 0.05) and the log‐likelihood (AIC, BIC, ABIC; smallest value) were used, with model retention decided on the basis of combinatorial rules advised by Hu and Bentler (1999).

## Results

### Descriptive statistics

Table S1 reports the descriptive statistics for the disability measurements at waves 1 and 7. Table S5 reports the correlations between measurements of pain and disability at each wave. The measures of pain were significantly associated with disability. Measures of disability correlated with age as well as with pain, particularly at wave 7. Table S6 reports how disability changed across the four identified trajectories among respondents with complete data at waves 1 and 7, including the magnitude of change in symptoms.

Table S5 reports the correlations between pain and domain of disability (mobility, ADL and IADL). The correlations between the domains of disability and pain remained consistent at different waves of the ELSA. These associations were stronger cross‐sectionally (mean *r* mobility = .486, ADL = 0.312, IADL = 0.260) and became slightly weaker over time (mean lag‐1 *r* mobility = .414, ADL = 0.272, IADL = 0.227). Across all waves, these associations were strongest for mobility (mean *r* = .395) and weaker for ADL (mean *r* = .256) for IADL (mean *r* = .210). The tables also show that the relationship between pain and a measure of disability (or vice versa) became weaker with increasing distance in wave between the two measures (a simplex structure).

### Homogenous growth models

Treating the sample as homogenous (Table 1), intercepts and slopes in all three domains significantly differed from zero or unity, respectively; cases with an average age and who are male reported more than zero difficulties in all three areas, and this increased over time. The intercepts for the mobility model varied by age (older age was associated with greater mobility problems) and sex (more problems among women), but not for the other areas. In all three models, age was associated with steeper slopes. That is, older respondents reported greater worsening in their everyday function. All of the models had good fit, according to indices based on transformations to the chi‐square statistic (CFI, TLI, RMSEA) and absolute fit (SRMR).

**Table 1:** Linear growth models looking at the changes in disability (adjusting for age and sex) in the English Longitudinal Study of Ageing when arthritis pain is treated as homogeneous (*n* = 888). Missing data are accounted for using a pattern mixture model

Effect	*b*	*SE*	*p*
Mobility χ^2^(63) = 97.711, *p* = .003, RMSEA = .025 (CI's 0.015–0.034), CFI = 0.989, TLI = 0.986, SRMR = 0.045, AIC = 17,091.661, BIC = 17,225.752, ABIC = 17,136.829			
Intercept:	1.948	.161	<.001***
Age (*z*)	0.209	.085	.014*
Sex	0.326	.166	.049*
Slope:	0.164	.032	<.001***
Age (*z*)	0.118	.020	<.001***
Sex	0.042	.036	.229
ADL χ^2^(63) = 94.806, *p* = .006, RMSEA = .024 (CI's 0.013–0.033), CFI = 0.984, TLI = 0.981, SRMR = 0.038, AIC = 11,179.015, BIC = 11,313.106, ABIC = 11,224.184			
Intercept:	0.451	.066	<.001***
Age (z)	0.051	.035	.136
Sex	−0.097	.068	.153
Slope:	0.049	.018	.007**
Age (z)	0.058	.011	<.001***
Sex	0.010	.020	.633
IADL χ^2^(63) = 130.845, *p* < .001, RMSEA = .035 (CI's 0.026–0.043), CFI = 0.966, TLI = 0.958, SRMR = 0.058, AIC = 11,054.160, BIC = 11,188.251, ABIC = 11,099.329			
Intercept:	0.315	.066	<.001***
Age (z)	0.049	.034	.157
Sex	0.041	.068	.552
Slope:	0.047	.018	.010*
Age (z)	0.097	.012	<.001***
Sex	0.039	.021	.057

Notes

ABIC = adjusted Bayesian information criterion; ADL = activities of daily living; AIC = Akaike information criterion; BIC = Bayesian information criterion; CFI = comparative fit index; IADL = instrumental activities of daily living; RMSEA = root mean square error of approximation; SRMR = standardized root mean squared residual; TLI = Tucker‐Lewis index. Unstandardized Coefficients.

*p < .05; **p < .01; ***p < .001.

### Heterogeneous growth models

**Figure 1:**
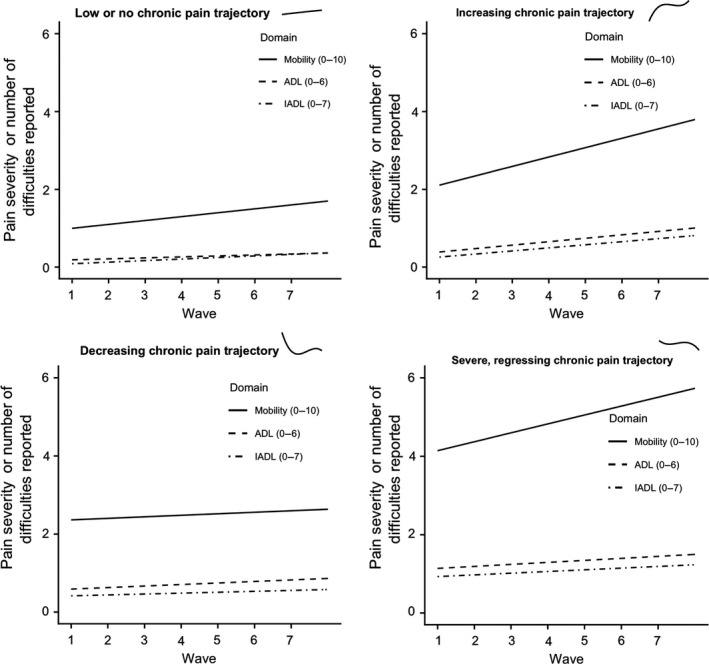
Levels of difficulty (in mobility, activities of daily living [ADL], and instrumental ADL [IADL]) at each wave for each of the four trajectories of pain. The pain trajectory over time is sub‐plotted next to the name of each trajectory.

Adding trajectories into the model in order to model heterogeneity indicated that this more complex model performed equally well on all measures of fit but performed much better based on indices derived from the log‐likelihood (AIC, BIC, ABIC). The growth models (Table 2, Figure 1) show that membership of any of the trajectories (apart from increasing chronic pain in IADL) was associated with higher baseline disability relative to the reference category. Membership of the ‘increasing chronic pain’ group was associated with greater growth in reporting disability for mobility and ADL, and those in the ‘severe, fluctuating chronic pain’ group reporting greater mobility complaints. Membership of the ‘decreasing chronic pain’ group was not associated with increases or decreases in disability. In all three domains, increasing age was associated with increasing reports of disability over time and greater reporting of disability at baseline. In addition, women reported fewer impairments in ADL at baseline and greater growth in IADL over time.

**Table 2:** Linear growth models looking at the effects of trajectory, age, and sex on changes in disability over the seven waves of the English Longitudinal Study of Ageing (*n* = 888). Missing data are accounted for using a pattern mixture model; trajectories are dummy coded (low/no chronic pain as the reference class)

Effect	*b*	*SE*	*p*
Mobility χ^2^(78) = 146.463, *p* < .001, RMSEA = .031 (CI's 0.023–0.039), CFI = 0.98, TLI = 0.975, SRMR = 0.043, AIC = 16,747.799, BIC = 16,910.624, ABIC = 16,802.647			
Intercept:	0.995	.159	<.001***
Increasing pain	1.114	.202	<.001***
Decreasing pain	1.369	.204	<.001***
Severe, fluctuating pain	3.148	.177	<.001***
Age (*z*)	0.301	.073	<.001***
Sex	0.239	.142	.092
Slope:	0.100	.033	.002**
Increasing pain	0.141	.042	.001**
Decreasing pain	−0.061	.046	.191
Severe, fluctuating pain	0.127	.041	.002**
Age (*z*)	0.113	.018	<.001***
Sex	0.040	.032	.202
ADL χ^2^(78) = 130.330, *p* = .002, RMSEA = .027 (CI's 0.019–0.036), CFI = 0.976, TLI = 0.970, SRMR = 0.038, AIC = 11,032.48, BIC = 11,195.306, ABIC = 11,087.329			
Intercept:	0.187	.070	.007**
Increasing pain	0.201	.089	.023*
Decreasing pain	0.408	.089	<.001***
Severe, fluctuating pain	0.956	.078	<.001***
Age (*z*)	0.077	.032	.016*
Sex	−0.131	.062	.034*
Slope:	0.025	.019	.189
Increasing pain	0.063	.024	.010*
Decreasing pain	0.014	.027	.606
Severe, fluctuating pain	0.026	.024	.277
Age (z)	0.056	.010	<.001***
Sex	0.010	.019	.582
IADL χ^2^(78) = 142.549, *p* < .001, RMSEA = .031 (CI's 0.022–0.038), CFI = 0.969, TLI = 0.961, SRMR = 0.050, AIC = 10,938.040, BIC = 11,100.865, ABIC = 10,992.888			
Intercept:	0.087	.073	.230
Increasing pain	0.170	.092	.066
Decreasing pain	0.336	.093	<.001***
Severe, fluctuating pain	0.848	.081	<.001***
Age (z)	0.071	.033	.033*
Sex	0.008	.065	.906
Slope:	0.040	.022	.068
Increasing pain	0.039	.027	.156
Decreasing pain	−0.017	.030	.574
Severe, fluctuating pain	0.003	.026	.911
Age (*z*)	0.096	.012	<.001*
Sex	0.041	.021	.049*

ABIC = adjusted Bayesian information criterion; ADL = activities of daily living; AIC = Akaike information criterion; BIC = Bayesian information criterion; CFI = comparative fit index; Decreasing = decreasing chronic pain; IADL = instrumental activities of daily living; Increasing = increasing chronic pain; RMSEA = root mean square error of approximation; Severe = severe regressing chronic pain; SRMR = standardized root mean squared residual; TLI = Tucker‐Lewis index. Unstandardized Coefficients.

*p < .05; **p < .01; ***p < .001.

Table S7 reports the proportions of each trajectory that reported their disability improving, staying the same or worsening from wave to wave. Table S8 and S9 report the amount of difficulties experienced in each domain within each trajectory at waves 1 and 7, respectively. There was a slight increase in disability over time in all trajectories. Increasing disability was least in the decreasing pain trajectory group, whereas the increasing pain trajectory group showed substantial increases in difficulty across each of the domains. People in the low or no chronic pain trajectory group reported statistically significant increases in disability in all three domains, and the severe pain group in two of the three.

## Discussion

When treating arthritic pain progression as homogeneous, the associations between pain and disability indicated that there was a moderate, positive relationship between increased pain and increased disability in all three domains of daily living. However, moving beyond this simple homogenous model and incorporating trajectories to explore the heterogeneous effects of pain over time revealed a more complex link between pain and disability. When adjusting for baseline disability, respondents whose pain became worse, or remained severe, had higher levels of disability relative to those who had minimal pain and remained stable. However, in the respondents whose pain improved, disability also remained high and did not improve correspondingly with improving pain. Importantly our findings suggest that disability remains a long‐term problem for people with arthritis, even when their pain improves.

It is not surprising that worsening pain is associated with greater reports of disability. Pain is a commonly reported cause for limiting activities, a key component of reporting disability (Verbrugge & Juarez, 2006), and it has been previously shown that trajectories of worsening disability in rheumatoid arthritis are associated with increasing pain (Norton *et al*., 2013). Similarly, studies of disability and pain in response to treatment show that patients with more limited function post‐intervention appear to be the same ones reporting greater pain (Lee *et al*., 2018; Lenguerrand *et al*., 2016).

The results of modelling heterogeneity suggest that improvements in pain do not necessarily bring improvements in disability related to everyday activities. Although pain is commonly cited as a cause of limitation in ADL, the alleviation of pain did not in all cases result in a reduction in disability. Respondents in the decreasing chronic pain trajectory did not report reducing disability. Despite reporting alleviated pain, this group also did not report improvements in their social and civic engagement (James *et al*., 2018). The results of this analysis suggest that reduced engagement in social life might occur because this group continues to report difficulties in ADL despite a reduction in pain. The items in the ELSA, which probe activities such as walking across a room, getting out of bed, eating or using the bathroom, indicate that these respondents still experience some severe difficulties. The most commonly reported difficulties in the whole sample were related to personal appearance and hygiene, dressing and bathing oneself (Tables S8 and S9; Gale, Cooper, & Aihie Sayer, 2015). The identified improvement in pain may have occurred because respondents adapted to changes in their circumstances by limiting their daily activities, and this then became their habitual way of living. While these respondents may be suffering less from the physical symptoms associated with arthritis, arthritis‐related disability may continue to have a profound effect upon their psychological well‐being. Respondents may report increased disability due to the substantial pain they report in the first three waves. Further work might benefit from looking at the mechanisms underlying pain in this ‘improving pain’ group and which (if any) interventions they took to improve their pain. This appears to be a group where rapid intervention is critical, as the effects of arthritis on daily living continue after the primary symptoms improve. Further attempts should be undertaken to identify these patients. Although pain is often identified as a cause for increasing difficulties with mobility and daily living, pain and disability were only moderately correlated. Previous research has shown that pain relief following joint replacement surgery for arthritis is not necessarily associated with increased physical activity (Almeida, Khoja, & Piva, 2018). People with disability due to pain might come to accept their lower level of activity, or may be fearful that activity might bring back the pain that they have previously experienced, and therefore do not necessarily increase activity after pain improves. While these analyses suggest pain is a major factor in disability, it further suggests that other factors are also at play and that studying the mechanisms underlying pain in this group and efficacious intervention approaches is crucial, in order to target behaviour change programmes that address disability.

With respect to models used in the health psychology literature (e.g., Health Beliefs Model), which aim to describe how psychological factors influence physiological, emotional and behavioural responses to illness, these results show clearly that patient heterogeneity needs to be considered and included. In this paper, we segment the sample in terms of pain/disease trajectories; however, here are many other characteristics that can be used across different diseases (e.g., early vs. last onset, chronicity, reactivity to treatment). We urge researchers to consider the potential heterogeneity of their samples and incorporate this into their theorizing and analyses.

There are some limitations associated with this analysis. Respondents were assigned to trajectories based on their most likely latent class. However, with latent class models each respondent has a varying posterior probability of being assigned to a class, and this analysis does not account for the corresponding uncertainty although the classification accuracy was high. The measures of disability (especially IADL) are also skewed, which means that observing reductions in disability is difficult because most respondents are experiencing minimal disability in the first place. As a general population sample, although it is generalizable to the wider population, respondents are likely to report lower levels of pain and disability than arthritis‐specific cohorts. However, similar trajectory structures have been identified in arthritis and general population cohorts (James *et al*., 2018).

These results highlight the strengths of segmenting a patient group that has different experiences of pain. Taken as a homogenous group, the models showed that disability progressed over time, that progression was associated with older age, and that there was a stable association between pain and disability over time. Segmenting the population by pain trajectories revealed that greater levels of disability were identified in those trajectories reporting greater pain at baseline. However, for some groups, especially those reporting improved pain, these changes in pain were not associated with improvements in disability. Thus, disabilities in an arthritic sample are not simply a linear function of currently experienced pain. There is an incongruent group who reported improving pain associated with worsening disability. This segment of around one‐fifth of the sample, despite showing minimal pain, experienced difficulties in their daily living that did not improve with their pain alleviating. As such, clinicians should not simply reason that if patients report improving pain that they will also experience improvements in other aspects of their lives. Thus, if a patient reports their pain is getting better they may still require help with daily living and these services should remain in place.

## Conflict of interests

All authors declare no conflict of interest.

## Funding

This project was supported by Versus Arthritis (grant number: 20777), previously Arthritis Research UK, and the National Institute for Health Research via the NIHR Nottingham Biomedical Research Centre. The funder had no role in the design of the study, data collection, analysis or interpretation, nor the writing of or the decision to submit the manuscript.

## Supporting information


**Table S1.** Disability items by domain.
**Table S2.** Descriptive statistics for the continuous variables modelled.
**Table S3.** Linear growth models looking at the effects of trajectory, age and sex on changes in disability over the seven waves of the ELSA excluding people known to have died on the basis of health records at Wave 5 (*n* = 719).
**Table S4.** Linear growth models looking at the changes in disability (adjusting for age and sex) in the ELSA when arthritis pain is treated as homogeneous and participants have covariate and disability data at all seven waves (*n* = 335).
**Table S5.** Pearson correlations between pain and mobility at different waves of the ELSA.
**Table S6.** Extent of disability in each trajectory at Waves 1 and 7.
**Table S7.** Levels of change (compared to previous wave) in each domain of disability from wave to wave for each of the four trajectories.
**Table S8.** Disability at Wave 1.
**Table S9.** Disability at Wave 7.
