# Supplementary material for: Trajectories of pain predict disabilities affecting daily living in arthritis
Source: Br J Health Psychol. 2019 Apr 7;24(3):485–96. doi: 10.1111/bjhp.12364 (PMC6916370; doi:10.1111/bjhp.12364)
Supplement: Supplementary file 1 — Table S1. Disability items by domain. Table S2. Descriptive statistics for the continuous variables modelled. Table S3. Linear growth models looking at the effects of trajectory, age and sex on changes in disability over the seven waves of the ELSA excluding people known to have died on the basis of health records at Wave 5 (n = 719). Table S4. Linear growth models looking at the changes in disability (adjusting for age and sex) in the ELSA when arthritis pain is treated as homogeneous and participants have covariate and disability data at all seven waves (n = 335). Table S5. Pearson correlations between pain and mobility at different waves of the ELSA. Table S6. Extent of disability in each trajectory at Waves 1 and 7. Table S7. Levels of change (compared to previous wave) in each domain of disability from wave to wave for each of the four trajectories. Table S8. Disability at Wave 1. Table S9. Disability at Wave 7. [file BJHP-24-485-s001.docx]

**Table S1.** *Disability items by domain*

| Mobility (10 questions) |
| --- |
| Walking 100 yards |
| Sitting for about two hours |
| Getting up from a chair after sitting for long periods |
| Climbing several flights of stairs without resting |
| Climbing one flight of stairs without resting |
| Stooping, kneeling or crouching |
| Reaching or extending your arms above shoulder level |
| Pulling or pushing large objects (like a living room chair) |
| Lifting or carrying weights over 10 pounds (like a heavy bag) |
| Picking up a 5 pence coin from a table |
| Activities of Daily Living (6 questions) |
| Dressing, including putting on shoes and socks |
| Walking across a room |
| Bathing or showering |
| Eating, such as cutting up your food |
| Getting in or out of bed |
| Using the toilet, including getting up or down |
| Instrumental Activities of Daily Living (7 questions) |
| Using a map to figure out how to get around in a strange place |
| Preparing a hot meal |
| Shopping for groceries |
| Making telephone calls |
| Taking medications |
| Doing work around the house or garden |
| Managing money, such as paying bills and keeping track of expenses |

**Table S2.** *Descriptive statistics for the continuous variables modelled*

| Measure | *M* | *SD* | Skew | Kurtosis | α |
| --- | --- | --- | --- | --- | --- |
| *Wave 1* |  |  |  |  |  |
| Mobility (0-10) | 2.537 | 2.576 | 0.976 | 0.025 | 0.82 |
| ADL (0-6) | 0.508 | 1.032 | 2.382 | 5.706 | 0.70 |
| IADL (0-7) | 0.484 | 1.041 | 2.697 | 8.538 | 0.71 |
| Age | 64.631 | 10.437 | 0.277 | -0.849 |  |
| *Wave 7* |  |  |  |  |  |
| Mobility (0-10) | 3.060 | 2.920 | 0.723 | -0.582 | 0.86 |
| ADL (0-6) | 0.662 | 1.347 | 2.519 | 6.169 | 0.82 |
| IADL (0-7) | 0.690 | 1.419 | 2.731 | 7.702 | 0.83 |

Abbreviations *ADL* Activities of daily living *IADL* Instrumental activities of daily living

**Table S3.** *Linear growth models looking at the effects of trajectory, age and sex on changes in disability over the seven waves of the ELSA excluding people known to have died on the basis of health records at Wave 5 (n = 719). Missing data is accounted for using a pattern mixture model, trajectories are dummy coded (low/no chronic pain as the reference class).*

| Effect | *b* | *SE* | *p* |
| --- | --- | --- | --- |
| *Mobility χ^2^(78) = 150.805, p < .001, RMSEA = .036 (CI’s .027 - .045), CFI = 0.977, TLI = 0.972, SRMR = 0.029, AIC = 14813.692, BIC = 14969.349, ABIC = 14861.380* | | | |
| Intercept: | 0.935 | 0.164 | <.001 *** |
| Increasing pain | 1.192 | 0.207 | <.001 *** |
| Decreasing pain | 1.269 | 0.223 | <.001 *** |
| Severe, fluctuating pain | 3.368 | 0.191 | <.001 *** |
| Age (z) | 0.187 | 0.085 | .028 * |
| Sex | 0.207 | 0.153 | .176 |
| Slope: | 0.092 | 0.033 | .005 ** |
| Increasing pain | 0.142 | 0.041 | .001 ** |
| Decreasing pain | -0.035 | 0.047 | .447 |
| Severe, fluctuating pain | 0.118 | 0.041 | .004 ** |
| Age (z) | 0.109 | 0.018 | <.001 *** |
| Sex | 0.047 | 0.032 | .145 |
| *ADL χ^2^(78) = 124.264, p = <.001, RMSEA = .029 (CI’s .019 - .038), CFI = 0.978, TLI = 0.972, SRMR = 0.047, AIC = 9558.571, BIC = 9714.218, ABIC = 9606.259* | | | |
| Intercept: | 0.180 | 0.069 | .009 ** |
| Increasing pain | 0.214 | 0.087 | .015 * |
| Decreasing pain | 0.351 | 0.094 | <.001 *** |
| Severe, fluctuating pain | 1.060 | 0.080 | <.001 *** |
| Age (z) | 0.071 | 0.036 | .047 * |
| Sex | -0.142 | 0.064 | .027 * |
| Slope: | 0.019 | 0.019 | .306 |
| Increasing pain | 0.058 | 0.023 | .014 * |
| Decreasing pain | 0.030 | 0.026 | .250 |
| Severe, fluctuating pain | 0.014 | 0.023 | .543 |
| Age (z) | 0.050 | 0.010 | <.001 *** |
| Sex | 0.017 | 0.018 | .339 |
| *IADL χ^2^(78) = 164.861, p <.001, RMSEA = .039 (Cis .031 - .048), CFI = 0.955, TLI = 0.944, SRMR = 0.040, AIC = 9063.276, BIC = 9218.923, ABIC = 9110.963* | | | |
| Intercept: | 0.035 | 0.063 | .574 |
| Increasing pain | 0.182 | 0.079 | .022 * |
| Decreasing pain | 0.332 | 0.085 | <.001 *** |
| Severe, fluctuating pain | 0.842 | 0.073 | <.001 *** |
| Age (z) | -0.050 | 0.033 | .123 |
| Sex | 0.030 | 0.058 | .609 |
| Slope: | 0.043 | 0.019 | .021 * |
| Increasing pain | 0.035 | 0.023 | .137 |
| Decreasing pain | -0.012 | 0.026 | .641 |
| Severe, fluctuating pain | 0.014 | 0.023 | .535 |
| Age (z) | 0.091 | 0.010 | <.001 *** |
| Sex | 0.026 | 0.018 | .142 |

*Abbreviations. ADL* Activities of daily living *IADL* Instrumental activities of daily living *Increasing* = Increasing chronic pain *Decreasing* = Decreasing chronic pain *Severe* = Severe regressing chronic pain. Unstandardized Coefficients.

Note - * = *p* < .05, **** *p* < .01, *** *p* < .001

**Table S4**. *Linear growth models looking at the changes in disability (adjusting for age and sex) in the ELSA when arthritis pain is treated as homogeneous and participants have covariate and disability data at all 7 waves (n = 335). Missing data is accounted for using a pattern mixture model.*

| Effect | *b* | *SE* | *p* |
| --- | --- | --- | --- |
| *Mobility χ^2^(33) = 68.336, p = <.001, RMSEA = .057 (CI’s .037 - .075), CFI = 0.982, TLI = 0.981, SRMR = 0.028, AIC = 9132.836, BIC = 9193.862, ABIC = 9143.108* | | | |
| Intercept: | 1.894 | 0.208 | <.001 *** |
| Age (z) | 0.108 | 0.149 | .470 |
| Sex | 0.318 | 0.249 | .202 |
| Slope: | 0.131 | 0.035 | <.001 *** |
| Age (z) | 0.088 | 0.025 | <.001 *** |
| Sex | 0.061 | 0.042 | .149 |
| *ADL χ^2^(33) = 87.858, p = <.001, RMSEA = .070 (CI’s .053 - .088), CFI = 0.959, TLI = 0.956, SRMR = 0.038, AIC = 5719.370, BIC = 5780.397, ABIC = 5729.643* | | | |
| Intercept: | 0.445 | 0.083 | <.001 |
| Age (z) | 0.020 | 0.060 | .735 |
| Sex | -0.102 | 0.100 | .307 |
| Slope: | 0.037 | 0.019 | .054 |
| Age (z) | 0.035 | 0.014 | .012 * |
| Sex | 0.007 | 0.023 | .761 |
| *IADL χ^2^(33) = 156.393, p <.001, RMSEA = .106 (CI’s .089 - 123), CFI = 0.900, TLI = 0.894, SRMR = 0.062, AIC = 5385.798, BIC = 5446.825, ABIC = 5396.071* | | | |
| Intercept: | 0.272 | 0.076 | <.001 *** |
| Age (z) | -0.060 | 0.055 | .274 |
| Sex | 0.077 | 0.091 | .399 |
| Slope: | 0.049 | 0.018 | .005 ** |
| Age (z) | 0.071 | 0.013 | <.001 *** |
| Sex | 0.010 | 0.021 | .642 |

*Abbreviations*. *ADL* Activities of daily living *IADL* Instrumental activities of daily living *Increasing* = Increasing chronic pain *Decreasing* = Decreasing chronic pain *Severe* = Severe regressing chronic pain. Unstandardized Coefficients. Please note the chi-square statistic has fewer degrees of freedom than the model reported in Table x because it has complete data so the pattern match was not required

Note - * = *p* < .05, **** *p* < .01, *** *p* < .001

**Table S5**. *Pearson correlations between pain and mobility at different waves of the ELSA.*

1. Pain and mobility

|  | W1M | W2M | W3M | W4M | W5M | W6M | W7M |
| --- | --- | --- | --- | --- | --- | --- | --- |
| W1P | 0.49 | 0.41 | 0.38 | 0.36 | 0.35 | 0.32 | 0.37 |
| W2P | 0.37 | 0.52 | 0.43 | 0.39 | 0.41 | 0.40 | 0.39 |
| W3P | 0.35 | 0.45 | 0.54 | 0.46 | 0.46 | 0.46 | 0.41 |
| W4P | 0.31 | 0.40 | 0.42 | 0.47 | 0.46 | 0.45 | 0.41 |
| W5P | 0.32 | 0.39 | 0.43 | 0.41 | 0.48 | 0.38 | 0.36 |
| W6P | 0.28 | 0.32 | 0.31 | 0.35 | 0.40 | 0.43 | 0.40 |
| W7P | 0.28 | 0.30 | 0.33 | 0.34 | 0.37 | 0.37 | 0.47 |

1. Pain and Activities of Daily Living (ADL)

|  | W1A | W2A | W3A | W4A | W5A | W6A | W7A |
| --- | --- | --- | --- | --- | --- | --- | --- |
| W1P | 0.39 | 0.30 | 0.27 | 0.21 | 0.26 | 0.21 | 0.15 |
| W2P | 0.32 | 0.34 | 0.27 | 0.25 | 0.26 | 0.25 | 0.18 |
| W3P | 0.29 | 0.31 | 0.33 | 0.28 | 0.27 | 0.30 | 0.26 |
| W4P | 0.26 | 0.29 | 0.30 | 0.33 | 0.25 | 0.30 | 0.19 |
| W5P | 0.22 | 0.21 | 0.27 | 0.29 | 0.30 | 0.26 | 0.19 |
| W6P | 0.22 | 0.25 | 0.19 | 0.20 | 0.26 | 0.24 | 0.23 |
| W7P | 0.23 | 0.21 | 0.20 | 0.24 | 0.30 | 0.22 | 0.25 |

1. Pain and Instrumental Activities of Daily Living (IADL)

|  | W1I | W2I | W3I | W4I | W5I | W6I | W7I |
| --- | --- | --- | --- | --- | --- | --- | --- |
| W1P | 0.32 | 0.27 | 0.22 | 0.18 | 0.20 | 0.16 | 0.15 |
| W2P | 0.28 | 0.32 | 0.22 | 0.12 | 0.25 | 0.20 | 0.15 |
| W3P | 0.20 | 0.30 | 0.27 | 0.15 | 0.21 | 0.22 | 0.17 |
| W4P | 0.26 | 0.25 | 0.23 | 0.23 | 0.24 | 0.20 | 0.12 |
| W5P | 0.19 | 0.23 | 0.24 | 0.27 | 0.28 | 0.12 | 0.10 |
| W6P | 0.19 | 0.21 | 0.16 | 0.16 | 0.27 | 0.12 | 0.18 |
| W7P | 0.20 | 0.20 | 0.17 | 0.16 | 0.28 | 0.20 | 0.27 |

*Note. M* = Mobility *, A* = Activities of daily living*, I* = Instrumental activities of daily living, *P* = Pain. All mobility and ADL correlations are significant at *p* <.01 uncorrected for multiple comparisons and corrected using Holm’s correction, and IADL correlations are significant corrected and uncorrected at *p* < .05.

**Table S6**. *Extent of disability in each trajectory at Waves 1 and 7.*

| Measure | Trajectory | Mean (SD) at W1 (n = 887) | Mean (SD) at W1 (n = 397) | Mean (SD) at W7 (n = 397) | Cohen’s *d* |
| --- | --- | --- | --- | --- | --- |
| Mobility | Low or none | 1.402 (1.787) | 0.959 (1.360) | 1.573 (2.080) | 0.349 *** |
|  | Increasing | 2.043 (2.010) | 2.024 (1.945) | 3.477 (2.734) | 0.611 *** |
|  | Decreasing | 3.190 (2.538) | 2.710 (2.472) | 3.194 (2.862) | 0.181 |
|  | Severe | 4.435 (2.886) | 4.154 (2.815) | 5.756 (2.659) | 0.585 *** |
| ADL | Low or none | 0.178 (0.580) | 0.111 (0.383) | 0.298 (0.957) | 0.257 ** |
|  | Increasing | 0.326 (0.732) | 0.214 (0.561) | 0.698 (1.423) | 0.445 ** |
|  | Decreasing | 0.687 (1.090) | 0.500 (0.882) | 0.887 (1.569) | 0.304 |
|  | Severe | 1.093 (1.434) | 1.064 (1.489) | 1.244 (1.564) | 0.118 |
| IADL | Low or none | 0.207 (0.686) | 0.094 (0.380) | 0.415 (1.245) | 0.349** |
|  | Increasing | 0.284 (0.796) | 0.274 (0.797) | 0.640 (1.226) | 0.353* |
|  | Decreasing | 0.619 (1.155) | 0.500 (1.020) | 0.855 (1.658) | 0.258 |
|  | Severe | 1.014 (1.365) | 0.808 (1.094) | 1.218 (1.617) | 0.297* |

*Abbreviation*s. *ADL* Activities of daily living *IADL* Instrumental activities of daily living.

*Note*. Asterisks proceeding Cohen’s *d* denote statistical significance using a paired samples t-test for W1 vs W7 (N = 397) - * = *p* < .05, ** *p* < .01, *** *p* < .001

**Table S7.** *Levels of change (compared to previous wave) in each domain of disability from wave to wave for each of the four trajectories*

| Domain | Trajectory | W2 | W3 | W4 | W5 | W6 | W7 |
| --- | --- | --- | --- | --- | --- | --- | --- |
| Mobility | Low/none | 0.25,0.39, 0.36 | 0.32, 0.41, 0.27 | 0.23, 0.52, 0.25 | 0.21, 0.45, 0.34 | 0.22, 0.47, 0.31 | 0.26, 0.51, 0.23 |
|  | Increasing | 0.22, 0.27, 0.51 | 0.36, 0.24, 0.4 | 0.3, 0.34, 0.36 | 0.23, 0.34, 0.43 | 0.37, 0.33, 0.31 | 0.25, 0.31, 0.43 |
|  | Decreasing | 0.42, 0.24, 0.34 | 0.35, 0.36, 0.29 | 0.31, 0.32, 0.37 | 0.24, 0.4, 0.37 | 0.31, 0.37, 0.32 | 0.24, 0.29, 0.47 |
|  | Severe | 0.28, 0.30, 0.43 | 0.4, 0.22, 0.37 | 0.42, 0.28, 0.29 | 0.28, 0.2, 0.52 | 0.34, 0.27, 0.39 | 0.36, 0.25, 0.4 |
| ADL | Low/none | 0.06, 0.84, 0.11 | 0.11, 0.83, 0.06 | 0.06, 0.82, 0.12 | 0.09, 0.82, 0.09 | 0.06, 0.82, 0.12 | 0.08, 0.8, 0.11 |
|  | Increasing | 0.1, 0.74, 0.17 | 0.17, 0.67, 0.16 | 0.14, 0.59, 0.27 | 0.17, 0.68, 0.16 | 0.12, 0.69, 0.19 | 0.17, 0.63, 0.2 |
|  | Decreasing | 0.2, 0.58, 0.22 | 0.18, 0.67, 0.15 | 0.14, 0.72, 0.14 | 0.06, 0.71, 0.22 | 0.15, 0.66, 0.19 | 0.07, 0.71, 0.22 |
|  | Severe | 0.23, 0.51, 0.26 | 0.32, 0.42, 0.26 | 0.25, 0.53, 0.22 | 0.23, 0.44, 0.33 | 0.24, 0.53, 0.22 | 0.22, 0.49, 0.29 |
| IADL | Low/none | 0.05, 0.85, 0.1 | 0.1, 0.8, 0.11 | 0.06, 0.81, 0.12 | 0.1, 0.79, 0.12 | 0.07, 0.78, 0.15 | 0.05, 0.83, 0.12 |
|  | Increasing | 0.09, 0.69, 0.22 | 0.15, 0.66, 0.19 | 0.14, 0.69, 0.17 | 0.13, 0.7, 0.18 | 0.18, 0.63, 0.18 | 0.12, 0.66, 0.22 |
|  | Decreasing | 0.17, 0.61, 0.22 | 0.17, 0.65, 0.18 | 0.14, 0.68, 0.18 | 0.08, 0.76, 0.16 | 0.09, 0.75, 0.16 | 0.14, 0.66, 0.2 |
|  | Severe | 0.2, 0.48, 0.32 | 0.28, 0.47, 0.25 | 0.28, 0.51, 0.21 | 0.21, 0.44, 0.35 | 0.17, 0.48, 0.27 | 0.27, 0.47, 0.26 |

*Note*: Proportions of cases reported as improving, staying the same, and worsening in that order, compared to their previous score. For example for W2 Low/none .25 = improving, 0.39 = stayed the same , 0.36 = worsening

**Table S8.** *Disability at Wave 1*

| Disability | All | Low or no chronic pain | Increasing chronic pain | Decreasing chronic pain | Severe chronic pain |
| --- | --- | --- | --- | --- | --- |
|  | Proportion Answering YES | | | | |
| *Mobility* |  |  |  |  |  |
| Walking 100 yards | 125 (14.2%) | 20 (5.2%) | 12 (8.5%) | 30 (20.4%) | 63 (29.4%) |
| Sitting for about 2 hours | 176 (19.9%) | 34 (8.9%) | 24 (17.0%) | 31 (21.1%) | 87 (40.7%) |
| Getting up from a chair after sitting for a long period | 319 (36.1%) | 77 (20.2%) | 45 (31.9%) | 70 (47.6%) | 127 (59.3%) |
| Climbing flights of stairs without resting | 388 (43.9%) | 108 (28.3%) | 53 (37.6%) | 78 (53.1%) | 149 (69.6%) |
| Climbing flight of stairs without resting | 153 (17.3%) | 27 (7.1%) | 11 (7.8%) | 38 (25.9%) | 77 (36.0%) |
| Stooping, kneeling or crouching | 425 (48.1%) | 120 (31.5%) | 57 (40.4%) | 96 (65.3%) | 152 (71.0%) |
| Extending arms above shoulder level | 128 (14.5%) | 22 (5.8%) | 19 (13.5%) | 24 (16.3%) | 63 (29.4%) |
| Pulling or pushing large objects | 194 (22.0%) | 39 (10.2%) | 21 (14.9%) | 42 (28.6%) | 92 (43.0%) |
| Lifting or carrying 10lb+ weights | 275 (31.1%) | 70(18.4%) | 36 (25.5%) | 53 (36.1%) | 116 (54.2%) |
| Picking 5p coin off table | 57 (6.5%) | 17 (4.5%) | 19 (13.5%) | 7 (4.8%) | 23 (10.7%) |
| *ADL* |  |  |  |  |  |
| Dressing | 153 (17.3%) | 27 (7.1%) | 18 (12.8%) | 36 (24.5%) | 72 (30.8%) |
| Walking across a room | 32 (3.6%) | 2 (0.5%) | 1 (0.7%) | 7 (4.8%) | 22 (9.4%) |
| Bathing or showering | 134 (15.2%) | 25 (6.6%) | 15 (10.6%) | 30 (20.4%) | 64 (27.4%) |
| Eating, including cutting food | 19 (2.2%) | 5 (1.3%) | 2 (1.4%) | 3 (2.0%) | 9 (3.8%) |
| Getting in or out of bed | 76 (8.6%) | 5 (1.3%) | 7 (5.0% | 17 (11.6%) | 47 (20.1%) |
| Using the toilet | 35 (4.0%) | 4 (1.0%) | 3 (2.1%) | 8 (5.4%) | 20 (8.5%) |
| *IADL* |  |  |  |  |  |
| Using a map to get around | 44 (5.0%) | 10 (2.6%) | 3 (2.1%) | 15 (10.2%) | 16 (6.8%) |
| Preparing a hot meal | 49 (5.5%) | 8 (2.1%) | 6 (4.3%) | 10 (6.8%) | 25 (10.7%) |
| Shopping for groceries | 96 (10.9%) | 15 (3.9%) | 8 (5.7%) | 22 (15.0%) | 51 (21.8%) |
| Making telephone calls | 23 (2.6%) | 4 (1.0%) | 1 (0.7%) | 3 (2.0%) | 9 (3.8%) |
| Taking medications | 20 (2.3%) | 2 (0.5%) | 0 (0%) | 6 (4.1%) | 12 (5.1%) |
| Doing house or garden work | 176 (19.9%) | 35 (9.2%) | 20 (14.2%) | 30 (20.4%) | 91 (38.9%) |
| Managing money i.e. bills and expenses | 25 (2.8%) | 5 (1.3%) | 2 (1.4%) | 5 (3.4%) | 13 (5.6%) |

*Note*. n’s = 889 – whole sample; 381 – low or no chronic pain; 141 – increasing chronic pain; 147 – decreasing chronic pain; 234 – severe chronic pain

**Table S9***. Disability at Wave 7*

| Disability | All | Low or no chronic pain | Increasing chronic pain | Decreasing chronic pain | Severe chronic pain |
| --- | --- | --- | --- | --- | --- |
|  | Proportion Answering YES | | | | |
| *Mobility* |  |  |  |  |  |
| Walking 100 yards | 91 (22.9%) | 14 (8.2%) | 18 (20.9%) | 21 (33.9%) | 38 (48.7%) |
| Sitting for about 2 hours | 62 (15.6%) | 7 (4.1%) | 17 (19.8%) | 7 (11.3%) | 31 (39.7%) |
| Getting up from a chair after sitting for a long period | 163 (41.1%) | 39 (22.8%) | 42 (48.8%) | 25 (40.3%) | 57 (73.1%) |
| Climbing flights of stairs without resting | 189 (47.6%) | 47 (27.5%) | 40 (46.5%) | 36 (58.1%) | 66 (84.6%) |
| Climbing flight of stairs without resting | 99 (24.9%) | 15 (8.8%) | 24 (27.9%) | 17 (27.4%) | 43 (55.1%) |
| Stooping, kneeling or crouching | 239 (60.2%) | 74 (43.3%) | 57 (66.3%) | 37 (59.7%) | 71 (91.0%) |
| Extending arms above shoulder level | 68 (17.1%) | 9 (5.3%) | 23 (26.7%) | 6 (9.7%) | 30 (38.5%) |
| Pulling or pushing large objects | 118 (29.7%) | 18 (10.5%) | 32 (37.2%) | 24 (38.7%) | 44 (56.4%) |
| Lifting or carrying 10lb+ weights | 139 (35.0%) | 35 (20.5%) | 33 (38.4%) | 21 (33.9%) | 50 (64.1%) |
| Picking 5p coin off table | 47 (11.8%) | 11 (6.4%) | 13 (15.1%) | 4 (6.5%) | 9 (11.5%) |
| *ADL* |  |  |  |  |  |
| Dressing | 84 (21.2%) | 20 (11.7%) | 20 (23.3%) | 16 (25.8%) | 28 (35.9%) |
| Walking across a room | 29 (7.3%) | 4 (2.3%) | 6 (7.0%) | 9 (14.5%) | 10 (12.8%) |
| Bathing or showering | 60 (15.1%) | 10 (5.8%) | 13 (15.1%) | 11 (17.7%) | 26 (33.3%) |
| Eating, including cutting food | 18 (4.5%) | 4 (2.3%) | 5 (5.8%) | 1 (1.6%) | 8 (10.3%) |
| Getting in or out of bed | 41 (10.3%) | 6 (3.5%) | 9 (10.5%) | 10 (16.1%) | 16 (20.5%) |
| Using the toilet | 31 (7.8%) | 7 (4.1%) | 7 (8.1%) | 8 (12.9%) | 9 (11.5%) |
| *IADL* |  |  |  |  |  |
| Using a map to get around | 30 (7.6%) | 9 (5.3%) | 4 (4.7%) | 5 (8.1%) | 12 (15.4%) |
| Preparing a hot meal | 38 (9.6%) | 10 (5.8%) | 9 (10.5%) | 6 (9.7%) | 13 (16.7%) |
| Shopping for groceries | 54 (13.6%) | 9 (5.3%) | 12 (14.0%) | 12 (19.4%) | 21 (26.9%) |
| Making telephone calls | 15 (3.8%) | 6 (3.5%) | 1 (1.2%) | 5 (8.1%) | 3 (3.8%) |
| Taking medications | 17 (4.3%) | 7 (4.1%) | 3 (3.5%) | 3 (4.8%) | 4 (5.1%) |
| Doing house or garden work | 101 (25.4%) | 24 (14.0%) | 24 (27.9%) | 17 (27.4%) | 36 (46.2%) |
| Managing money i.e. bills and expenses | 19 (4.8%) | 6 (3.5%) | 2 (2.3%) | 5 (8.1%) | 6 (7.7%) |

*Note*. n’s = 397 – whole sample; 171 – low or no chronic pain; 86 – increasing chronic pain; 62 – decreasing chronic pain; 78 – severe chronic pain
